# Visualization of Gallbladder with In-111 Octreotide Scan

**DOI:** 10.4274/mirt.07108

**Published:** 2015-06-17

**Authors:** Filiz Özülker, Tamer Özülker, M. Tarık Tatoğlu, Aysun Küçüköz Uzun

**Affiliations:** 1 Okmeydanı Training and Research Hospital, Clinic of Nuclear Medicine, İstanbul, Turkey

**Keywords:** In-111-Octreotide, gallbladder, neuroendocrine tumors

## Abstract

A 54-year-old woman underwent octreotide scintigraphy for evaluation of neuroendocrine tumor recurrence. The images demonstrated unusual uptake in gallbladder area in addition to physiologic uptake at other tissues. Whole-body planar and SPECT imaging were repeated after fatty meal ingestion at 28 hours in order to figure out whether this activity was physiologic or not. Since the unusual uptake in the gallbladder was still detected at these images, additional images were obtained 72 hours after radionuclide injection. The activity in the gallbladder disappeared at these images revealing the physiologic nature of this unusual accumulation.

## Abstract (tr)

Elli dört yaşında bayan hastaya, nöroendokrin tümörün olası rekürrensinin saptanması amacıyla oktreotid sintigrafisi uygulandı. Görüntülerde, diğer dokulardaki fizyolojik tutulumlara ek olarak safra kesesi bölgesinde olağandışı tutulum gözlendi. Safra kesesindeki tutulumun fizyolojik olup olmadığının anlaşılabilmesi için 28. saatte yağlı yemek yenmesi sonrası tüm-vücut planar ve SPECT görüntüleri tekrarlandı. Safra kesesi bölgesindeki olağandışı aktivitenin bu görüntülerde de devam etmesi üzerine radyonüklidin enjeksiyonundan sonra 72. saatte görüntüler tekrarlandı. Bu görüntülerde safra kesesindeki aktivitenin kaybolması bu olağandışı tutulumun fizyolojik karakterde olduğunu gösterdi.

## INTRODUCTION

In-111 octreotide scan is widely used in the evaluation of neuroendocrine tumors and physiologic accumulation of the activity might lead to misinterpretation (1). Herein, we present a patient with neuroendocrine tumor who was imaged with In-111 octreotide and showed unusual uptake in the gallbladder.

## CASE REPORT

A 54-year-old woman was referred to our department of nuclear medicine for In-111 octreotide (Octreo Scan, Mallinckrodt Medical, Petten, the Netherlands) scanning. The patient had undergone an operation for neuroendocrine carcinoma 8 months ago and octreotide scintigraphy was planned with the purpose of detection of any possible recurrences.

**Figure 1:**
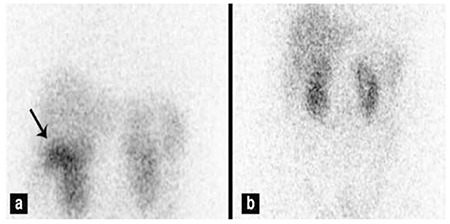
In-111 octreotide scan showing accumulation of activity in the liver (arrow) on planar image obtained 24 hour after injection (a), planar image obtained 72 hours after injection of radionuclide showing clearance of the activity (b)

**Figure 2:**
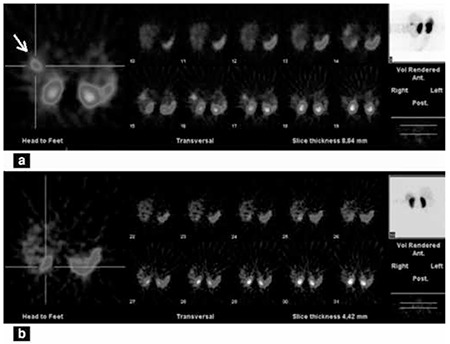
In-111 octreotide SPECT image at 24 hours showing activity in the liver on axial slice (a), 72 hours SPECT image showing clearance of the uptake (b)

Octreotide scintigraphy was performed and whole-body planar and SPECT were acquired at 4 and 24 hours after administration of 5 mCi (185 MBq) In-111 octreotide. The images demonstrated physiologic uptake at the liver, spleen, kidneys and bladder along with an unusual uptake at the location of gallbladder. Whole-body planar and SPECT imaging were repeated after fatty meal ingestion at 28 hours in order to differentiate pathological activity and physiologic uptake in the gallbladder. The unusual uptake in the gallbladder was still detected at these images; therefore additional images were obtained 72 hours after radionuclide injection. The activity in the gallbladder disappeared at these images indicating the physiologic nature of this unusual accumulation (Figure 1 and Figure 2).

## LITERATURE REVIEW AND DISCUSSION

In-111 octreotide has been widely used for 20 years for scintigraphic localization of primary and metastatic neuroendocrine tumors that express somatostatin receptors. Normal physiologic distribution of In-111 octreotide includes faint visualization of thyroid, pituitary gland and marked increased uptake in the liver, spleen, kidneys and bladder. Although rarely encountered, false-positive studies have been reported due to increased activity at nonmalignant pathologies such as Paget’s disease, parathyroid adenoma, cholecystitis, thrombus, abscess, infection, pulmonary fibrosis, pleural plaques and uterine myomas (2,3,4,5,6,7,8,9). Only a few cases in the literature have reported increased In-111 octreotide activity in the gallbladder (10,11,12,13,14). The unusual accumulation of In-111 octreotide in benign pathologies may be explained partially by the presence of somatostatin receptors in activated leukocytes during chronic inflammatory processes (15,16). After injection, In-111 octreotide is rapidly cleared by the kidneys (85% of the injected dose is recovered in the urine by 24 hours) and approximately 2% of the injected dose undergoes hepatobiliary excretion. Functional impairment is another possible reason for gallbladder visualization due to a delay in radionuclide excretion. Turner et al. showed that gallbladder motility is impaired in patients receiving long-acting somatostatin analogue and there might be an association with octreotide treatment and gallstone development (17). In our case a gallbladder inflammation that could explain such an uptake was not identified. Krausz et al. encountered unusual uptake in the gallbladder in 3 patients who underwent In-111 octreotide scintigraphy, and this activity disappeared after a fatty meal. It is also advised to administer a laxative on the day before injection, especially when the abdomen is the area of interest (18). Kurtaran et al. proposed that this problem might be prevented by performing a dual tracer SPECT imaging using In-111 octreotide and hepatobiliary agent, 99mTc-trimethyl-brom-imino-diacetic acid (TBIDA) with dual energy window sets (13). In our case the activity in the gallbladder persisted even after the patient had a fatty meal, but it disappeared in late images at 72 hours after injection.

We concluded that the images in In-111 octreotide scintigraphy should not be obtained in the fasting state and if significant uptake is seen in the gallbladder area, late images up to 72 hours should be acquired.

## References

[1] Biersack HJ, Briele B, Qian L, Mekkawy MA, Shih WJ (1992). The role of nuclear medicine in oncology. Ann Nucl Med.

[2] Kang S, Mishkin FS (1999). Visualization of Paget’s disease during somatostatin receptor scintigraphy. Clin Nucl Med.

[3] Servaes S, El-Haddad G, Zhuang H (2008). Intense octreotide activity in a thrombus. Clin Nucl Med.

[4] Lebtahi R, Moreau S, Brauner M, Soler P, Marchal J, Raguin O, Gruaz-Guyon A, Reubi JC, Le Guludec D, Crestani B (2006). Increased uptake of 111 In-octreotide in idiopathic pulmonary fibrosis. J Nucl Med.

[5] Lonneux M, Jamar F, Pauwels S (1998). Uptake of In-111 pentetreotide by pleural plaques. Clin Nucl Med.

[6] Stoffel M, Jamar F, Decoster P, Beckers C, Pauwels S (1996). Increased uptake of indium-111 pentetreotide up to 10 years after external thoracic irradiation: report of two cases. Eur J Nucl Med.

[7] Valero M, Boan JF, Rodriguez-Spiteri N, Torre W, Richter JA (2006). False positive In-111 DTPA octreotide scintigraphy resulting from lung abscess in a patient with metastatic neuroendocrine pancreatic neoplasm. Clin Nucl Med.

[8] Yenson T, Khalil T, Larcos G (2006). Pneumonia can cause 111 Indium octreotide uptake. Aust Radiol.

[9] Luisa M (2010). Mena, Francisco Martín, Ignacio Jime´nez. In-111 Pentetreotide Uptake in a Uterine Myoma. Clin Nucl Med.

[10] Niederkohr RD, McDougall IR (2007). Incidental gallbladder visualization on nonhepatobiliary nuclear medicine studies: case series and review of the literature. Clin Nucl Med.

[11] Gedik GK, Kiratli PO, Erbas B (2006). Visualization of gallbladder with In-111 labeled octreotide in post prandial state.

[12] Krausz Y, Shibley N, De Jong RB (1994). Gallbladder visualization with In-111 labeled octreotide.

[13] Kurtaran A, Ofluoglu S, Traub T, Tribl B, Speiser P, Grabenwöger F, Schima W, Dudczak R, Virgolini I (2000). An Unusual Visualization of the Gallbladder by Somatostatin Receptor (SSTR) Scintigraphy: Usefulness of Hepatobiliary Imaging for Differential Diagnosis. Am J Gastroenterol.

[14] Karaçavuş S, Kula M, Cihan KZ, Unlühızarcı K, Tutuş A, Bayram F, Coban G (2012). Octrotide uptake in parathyroid adenoma. Mol Imaging Radionucl Ther.

[15] Weinstock JV, Elliott DE (2000). The somatostatin immunoregulatory circuit present at sites of chronic inflammation. Eur J Endocrinol.

[16] Elliott DE, Li J, Blum AM, Metwali A, Patel YC, Weinstock JV (1999). SSTR2A is the dominant somatostatin receptor subtype expressed by inflammatory cells, is widely expressed and directly regulates T cell IFN-gamma release. Eur J Immunol.

[17] Turner HE, Lindsell DRM, Vadivale A, Thillainayagam AV, Wass JA (1999). Differing effects on gall-bladder motility of lanreotide SR and octreotide LAR for treatment of acromegaly. Eur J Endocrinol.

[18] Balon HR, Goldsmith SJ, Siegel BA, Silberstein EB, Krenning EP, Lang O, Donohoe KJ (2001). Procedure guideline for somatostatin receptor scintigraphy with (111)In-pentetreotide. J Nucl Med.

